# Effectiveness and cost-effectiveness of The Daily Mile on childhood weight outcomes and wellbeing: a cluster randomised controlled trial

**DOI:** 10.1038/s41366-019-0511-0

**Published:** 2020-01-28

**Authors:** Katie Breheny, Sandra Passmore, Peymane Adab, James Martin, Karla Hemming, Emma R. Lancashire, Emma Frew

**Affiliations:** 1 Institute of Applied Health Research, University of Birmingham, Birmingham, B15 2TT UK; 2 Bristol Medical School, University of Bristol, Bristol, BS8 1NU UK; 3 Services for Education, Birmingham, B7 4AX UK

**Keywords:** Risk factors, Cardiovascular diseases

## Abstract

**Background:**

The Daily Mile is designed to increase physical activity levels with children running or walking around school grounds for 15-min daily. It has been adopted by schools worldwide and endorsed as a solution to tackle obesity, despite no robust evidence of its benefits. We conducted a cluster randomised controlled trial to determine its clinical and cost-effectiveness.

**Methods:**

Forty schools were randomly assigned (1:1) to either the Daily Mile intervention or control group in which only the usual school health and wellbeing activities were implemented. The primary outcome was BMI *z*-score (BMIz) at 12 months follow-up from baseline, with planned subgroup analysis to examine differential effects. Primary economic analysis outcome was incremental cost per Quality-Adjusted-Life-Year (QALY) gained.

**Results:**

Using a constrained randomisation approach, balanced on school size, baseline BMIz and proportion of pupils eligible for free school meals, 20 schools were allocated to intervention (*n* = 1,153 participants) and 20 to control (*n* = 1,127); 3 schools withdrew (2 intervention, 1 control). At 12 months, BMIz data were available for 18 intervention schools (*n* = 850) and 19 control schools (*n* = 820 participants). Using intention-to-treat analysis the adjusted mean difference (MD) in BMIz (intervention − control) was −0.036 (95% CI: −0.085 to 0.013, *p* = 0.146). Pre-specified subgroup analysis showed a significant interaction with sex (*p* = 0.001) suggesting a moderate size benefit of The Daily Mile in girls (MD −0.097, 95% CI −0.156 to −0.037). This was consistent with the exploratory economic results that showed The Daily Mile to be highly cost-effective in girls (£2,492 per QALY), but not in boys, and overall to have a 76% chance of cost-effectiveness for the whole sample, at the commonly applied UK threshold of £20,000 per QALY.

**Conclusions:**

Overall the Daily Mile had a small but non-significant effect on BMIz, however, it had a greater effect in girls suggesting that it might be considered as a cost-effective component of a system-wide approach to childhood obesity prevention.

## Introduction

Childhood obesity is a major public health problem. Worldwide, around one in five children aged 5–19 years are either overweight or living with obesity [1]. In response to this global epidemic, the World Health Assembly adopted the ‘WHO global action plan for prevention and control of non-communicable diseases’ including halting childhood obesity rates by 2025 [2]. Children living with obesity are more likely to be overweight in adulthood [3], with associated greater risk of a range of chronic diseases and premature death [4]. Obesity also has a significant economic burden leading to costs equivalent to 2.8% of global gross domestic product [5]. The causes of the energy imbalance responsible for excess weight gain are the result of several interacting factors; including genetics, poor diet and physical inactivity [6]. The WHO global action plan for obesity guides action at the national, regional and local level and, as children spend a large proportion of their waking time in school, this has been cited as an appropriate setting to deliver childhood obesity prevention interventions. This is supported by findings from high-quality systematic reviews [7, 8] that suggest school-based interventions may be effective, although the heterogeneity of the included studies precludes recommendations on the exact intervention.

The ‘Daily Mile’ is a school-based intervention that involves children doing an additional 15 min of physical activity every school day, over and above national curriculum physical education (PE) and timetabled break times [9]. Based mainly on anecdotal reports of benefit in terms of reducing obesity and improving academic attainment, as well as seemingly low cost, over 6000 schools and nurseries (pre-school centres) worldwide [10] have adopted the intervention. Furthermore the UK Government called for all primary schools to adopt initiatives such as The Daily Mile within their updated Childhood Obesity Plan [11]. However published evaluations of the intervention are limited to one small school pilot non-randomised study, which suggested a minimal reduction in body fat in children following introduction of The Daily Mile [12]. A Cochrane review of previous school based physical activity interventions has shown that whilst overall such programmes increase physical activity levels during the school period and reduce the time children are sedentary, their impact on overall physical activity levels is relatively small (between 5 and 45 min additional activity per week), and there was no evidence that the programmes reduce levels of obesity [8].

In this cluster randomised controlled trial (RCT), we aimed to evaluate the clinical- and cost-effectiveness of The Daily Mile for obesity prevention in children, when compared with usual health and wellbeing activities in schools.

## Subjects and methods

### Study design

This was a rapid, pragmatic cluster RCT in 40 state-funded primary and junior schools (clusters) located in the south of Birmingham, England. Birmingham is the second largest city in England and it is ranked the 3rd most deprived core city after Liverpool and Manchester [13]. Deprivation has a strong association with childhood obesity [14]. Ethics approval was obtained from the University of Birmingham Research Ethics Committee (reference number 16–0064). The trial was conducted according to a published protocol that was implemented without changes [15] and this paper follows the extended CONSORT guidelines for the reporting of cluster randomised trials. This study is registered with ISRCTN (12698269).

### Participants

All state-funded primary and junior schools (children aged 4–11 years) located in South Birmingham with at least 20 children in school years 3 (aged 7–8 years) and 5 (aged 9–10 years) at baseline were eligible to participate. Intervention schools were encouraged to implement The Daily Mile in all year groups, however outcome measurements were obtained only from children in years 3 and 5. Children with a disability preventing them from running or walking for 15 min were excluded, as were those unable to have their height and weight measured at baseline. Written consent for outcome measures was obtained from parents/guardians and verbal assent obtained from eligible children. Baseline measurements were completed prior to randomisation.

### Randomisation and masking

Schools were randomised to either the control or intervention arm using a 1:1 allocation ratio. To minimise imbalance in important covariates, an independent statistician used a constrained randomisation based algorithm [16] in a statistical package (R, version 3.2.2). The algorithm generates a balance statistic, based upon the imbalance of the pre-specified school level covariates (baseline school average BMI *z*-score (BMIz), percentage of pupils eligible for free school meals and school size) to the intervention and control arms. The balance statistic is calculated for 1000 possible allocations. After the balance statistics are generated, a set of optimal allocations are produced for those allocation who exhibit <10% imbalance. The final allocation was chosen at random from this set of optimal allocations. Due to the nature of the intervention it was not possible to mask school staff, children, family members and project staff to the intervention allocation, however, all research staff undertaking the physical measurements were blinded.

### The intervention

Implementation was pragmatic to fit around the school annual timetable including school vacation. Schools were provided with information regarding The Daily Mile and directed to The Daily Mile website [10] for further guidance and resources. The intervention was carried out in lesson time at a time to suit each class during the school day, children left the classroom to run or walk around a pre-defined route within the school grounds for 15 min (on average equivalent to a distance of around 1 mile). The intervention was carried out in all but severe adverse weather conditions and required no change of clothing or footwear and was not a substitute for PE or break-times. Whilst advised as a daily activity, the frequency and duration were at the class teacher’s discretion. Although intervention development was not theoretically informed a priori, several characteristics were in keeping with established behaviour change theory and evidence-based techniques. These include promotion of autonomy (empowering children to set their pace and teachers to choose when and how to run the programme), engendering a sense of belonging (inclusive of all children, connecting with others and their teachers) and promoting achievement of competence (simple skill that is easily achieved and sufficiently challenging). Furthermore, class teachers delivered the intervention and were permitted to adapt it for implementation, using motivational material such as certificates, or using it to facilitate learning within another subject area such as Maths.

### Comparator

The control arm received no active intervention. Schools continued with usual health and wellbeing activities and were requested not to implement new health or physical activity initiatives for the duration of the study. Currently the amount of physical activity that primary schools should provide in the UK is not mandated, however at least 2 h a week is recommended by the Office for Standards in Education, Children’s Services and Skills (UK OFSTED [17]). More recently, the UK Government Childhood Obesity Strategy states that schools should provide 30 min of moderate to vigorous activity daily [11].

### Outcomes

All outcome measures were collected at baseline and 12 months. Selected outcomes were also collected at 4-month follow-up. The primary outcome for clinical effectiveness was BMIz at the 12-month follow-up. Secondary outcomes collected at 4 months were BMIz, fitness and body fat percentage. Secondary outcomes at 12-months were fitness, body fat percentage, child-reported quality of life, child-wellbeing and teacher-rated academic attainment (overall attainment and attainment in maths, reading and writing).

Full details of data collection methods are reported in the trial protocol [15]. BMIz were calculated using LMS growth software [18] and based upon age and sex-specific British 1990 growth reference data [19]. Fitness was measured using the British Athletics Linear Track Test [20] whereby children were encouraged to run as far as they could in two minutes on a pre-measured 50 m linear track. Self-reported quality of life was measured using the Child Health Utility 9 Dimension (CHU9D), a generic measure designed for children aged between 7 and 11 years including domains about being worried, sad, in pain, tired, annoyed, having problems with school work, daily routine and being able to join in with activities [21]. The CHU9D generates a utility score on a scale of zero to one representing a state equivalent to death and full-health, respectively. Child well-being was measured using the Middle Years Development Instrument (MDI), which measures social and emotional health and wellbeing in middle childhood (6–12 years). A wellbeing sum score was generated by adding items used to derive the MDI Wellbeing Index, which classifies children as having low, medium/high or very high wellbeing [22]. The score ranges from 15 to 75, with higher scores reflecting higher wellbeing. Both the CHU9D and MDI were completed electronically, under the supervision of school teaching staff, who were given instructions from the research team on how to do this. Trained research staff collected the physical measurements. The Linear track test was conducted by the PE school staff, using a standardised protocol. Measurements of academic attainment were obtained by asking teachers to rate each child’s performance in maths, reading and writing according to their age-related expectations [23]. Ratings were on a five-point scale, ranging from below expected to higher than expected. A total attainment score was obtained by summing the ratings for the three subjects.

### Study implementation

The intervention was delivered over 12 months (April 2017–March 2018). Baseline measures were collected in February/March 2017 and schools were randomised in April 2017. First follow-up data were collected for selected outcomes at 4 months after baseline (July 2017), prior to the 6-week summer vacation. At this first follow up point, outcomes collected were limited to items needed for the BMIz, bodyfat percentage and fitness (linear track test) to limit the data collection burden for schools. Final follow-up data were collected for all outcomes, 12 months after baseline (March 2018). During this 12-month period, control schools were asked to not change any activities.

### Statistical analysis

Sample size was calculated based on the primary outcome (BMIz) to detect a between group (control versus intervention) BMIz difference of 0.125 (clinically important difference for obesity prevention [24]) with greater than 90% power, anticipating a follow-up sample of 2000 participants across 40 schools (50 children per school). The trial was powered assuming a likely estimate of the intracluster correlation coefficient (ICC) of 0.04 and an estimated correlation between baseline and follow-up measurements of 0.9. The study power was robust to changes in both of these correlation estimates. The study had a greater than 80% power for values of the ICC between 0.001 and 0.1, and for any value of the correlation between baseline and follow-up measurements greater than 0.8. As the variation between cluster sizes was expected to be minimal, no allowance was made for cluster size variation. The sample size was calculated using the clustersampsi function in STATA version 13 [25, 26].

Participants with outcome assessment data were analysed according to allocated arm, irrespective of whether or not the participants adhered to the intervention. Analysis of the primary outcome used a mixed-effect linear regression model with 12-month BMIz as the dependent variable and trial arm and baseline BMIz as independent variables. School (cluster) was included as a random effect. In partially adjusted models, the analysis was adjusted for covariates used in the constrained randomisation (baseline school mean BMIz, percentage of children eligible for free school meals and school size) [27, 28]. In the further adjusted models, an additional adjustment was made for pre-specified pupil level covariates (age, sex, and ethnicity) and school level deprivation (UK Index of Multiple Deprivation score based on school postcode). Significance was considered at the 5% level.

Analysis of secondary outcomes replicated the partially and further adjusted models used for the primary outcome. Body fat, fitness, quality of life (CHU9D) and wellbeing (MDI) were analysed using mixed-effect linear models. For measures of academic attainment, participants were categorised as performing below or at/above the expected level for their age. To estimate risk ratios for binary outcomes, mixed effects Poisson models with robust standard errors were fitted. Due to software limitations in STATA (no binomial model in mixed model function that allows for clustering), estimates of the risk difference were calculated by fitting a generalised estimating equation, assuming a binomial distribution, with an identity link. Estimates of the ICC were calculated for the primary outcome as a ratio of variances after fitting a mixed-effect linear model. Planned subgroup analyses, using interaction tests, assessed differential treatment effects by sex, year group, baseline BMI, deprivation level and ethnic mix of the school. Models were fitted to each subgroup separately to estimate the treatment effect in each group. A model was also fitted to the data with an interaction between the subgroup and the treatment arm, to assess whether there is evidence of a differential treatment effect.

Multiple imputation was conducted on the following secondary outcomes: quality-of-life and wellbeing. Imputation models included child (sex, age, ethnicity and year group) and school level covariates (school size, free school meal eligibility and school average BMIz) and all covariates used in the analysis (trial arm, baseline individual and school outcomes). School (cluster) was included as a fixed effect [29]. Thirty imputed datasets were created, analysis conducted on each dataset and combined to form one set of results using Rubin’s rules. All analyses were done using Stata version 13. STATA functions utilised included ‘mixed’ for hierarchical linear regression, ‘mepoisson’ for Poisson models and ‘xtgee’ for binomial models.

### Economic analysis

We conducted a within trial cost-utility analysis, from a public sector perspective based on quality-adjusted life years (QALYs). Additional detail regarding the economic evaluation is provided in the Supplementary Appendix. Other economic evaluation findings that broadens the framework for analysis will be reported elsewhere. The costs of the intervention were assumed to be only the value of the teacher’s time from supervision, any other costs were judged to be neglible and included items such as classroom displays, certificates and enhanced first-aid kits. The control schools were assumed to have zero cost and therefore the focus was on the cost from implementing The Daily Mile, in addition to ‘usual activities’. All costs are reported in 2017 GBP prices. Annual intervention cost per child was estimated based on the average pupil/teacher ratio of 27:1. QALYs were estimated from the CHU-9D data, using the UK tariff set [30]. The between arm difference in costs and QALYs at 12 months was calculated to produce an incremental cost-effectiveness ratio (ICER). To explore the uncertainty around the ICER, a probabilistic sensitivity analysis was applied using bootstrapping and a cost-effectiveness acceptability curve was constructed that shows the probability of cost-effectiveness at difference cost per QALY thresholds. In the UK, interventions are deemed cost-effective if the cost per additional QALY gained is less than £20,000 per QALY [31, 32]. As well as the whole sample, the analysis was conducted separately for boys and girls.

## Results

**Fig. 1:**
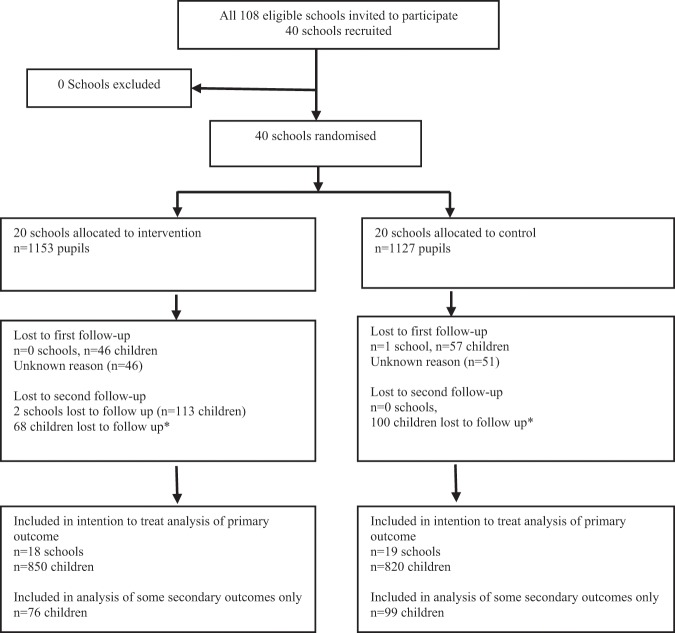
Participant flow. *Reason for individual children lost to follow-up is unknown because loss might be due to absence (illness) on the day of measurements or being out of the school classroom for an unknown reason at the time of measurements.

Participant flow through the study is presented in Fig. 1. Of 108 eligible schools invited to participate, 40 schools were included in the study. Parental consent for measurements was obtained from 2280 children at baseline. Three schools dropped out over the course of the study. Two schools (one intervention and one control) dropped out due to a change in headteacher, the third (intervention) school dropped out as it amalgamated with a nearby secondary school. Demographic and clinical characteristics of participants at schools that dropped out were similar to those completing the study. No adverse events were reported. Baseline characteristics and outcome measures were well balanced between the intervention and control arms (Table 1), although children in the control arm were slightly less likely to live in deprived areas (IMD quintiles 3–5) and fewer were in the White ethnic group.

**Table 1:** Baseline characteristics of children and schools participating in The Daily Mile study overall and by trial arm.

Characteristics	Intervention arm	Control arm	Total
**Demographic**	*n* = 1153	*n* = 1127	*n* = 2280
Sex:			
Female	549 (47.6)	534 (47.5)	1083 (47.5)
Male	604 (52.4)	591 (52.5)	1195 (52.5)
Not known^c^	0	2	2
Mean (SD) age (years)	8.8 (1.1)	8.8 (1.0)	8.9 (1.0)
Ethnicity:			
White British	614 (53.3)	559 (50.0)	1173 (51.5)
South Asian	186 (16.2)	183 (16.3)	369 (16.2)
Black African Caribbean	88 (7.6)	103 (9.2)	191 (8.4)
Other/not specified	264 (22.9)	279 (24.8)	543 (23.9)
Not known^c^	1	3	4
Deprivation fifth^a^:			
1 (most deprived)	575 (49.9)	621 (55.1)	1196 (52.5)
2	459 (39.8)	169 (15.0)	628 (27.5)
3	59 (5.1)	222 (19.7)	281 (12.3)
4	60 (5.2)	58 (5.2)	118 (5.2)
5 (least deprived)	0 (0.0)	57 (5.1)	57 (2.5)
**Anthropometric**			
BMI z score:	*n* = 1077	*n* = 1028	*n* = 2105
Mean (SD) BMI z score	0.37 (1.2)	0.38 (1.2)	0.38 (1.3)
Body fat %:	*n* = 1075	*n* = 1027	*n* = 2102
Mean (SD)	21.9 (7.1)	21.8 (6.9)	21.8 (7.0)
Weight status^b^:	*n* = 1077	*n* = 1028	*n* = 2105
Underweight (≤2nd centile)	16 (1.5)	27 (2.6)	43 (2.0)
Healthy weight (>2nd centile and <85th centiles)	728 (67.6)	684 (66.5)	1412 (67.1)
Overweight (≥85th centile and <95th centiles)	211 (19.6)	197 (19.2)	408 (19.4)
Obese (≥95th centile)	122 (11.3)	120 (11.7)	242 (11.5)
Not known^c^	76	99	175
**Physical activity**			
Linear track test (m):	*n* = 702	*n* = 639	*n* = 1341
Mean (SD)	351 (105)	346 (81)	348 (95)
**Quality of life and Wellbeing**			
CHU-9D utility score:	*n* = 801	*n* = 751	*n* = 1552
Mean (SD)	0.83 (0.22)	0.84 (0.16)	0.84 (0.16)
Middle Years Instrument Wellbeing sum score:	*n* = 801	*n* = 751	*n* = 1552
Mean (SD)	58.5 (10.9)	57.8 (11.0)	58.2 (11.0)
**Academic attainment**^d^			
Maths:	*n* = 628	*n* = 631	*n* = 1259
Mean (SD)	2.4 (1.2)	2.5 (1.1)	2.5 (1.2)
Reading:	*n* = 629	*n* = 629	*n* = 1258
Mean (SD)	2.5 (1.2)	2.5 (1.2)	2.5 (1.2)
Writing:	*n* = 629	*n* = 634	*n* = 1263
Mean (SD)	2.2 (1.1)	2.3 (1.2)	2.3 (1.1)
Overall academic attainment score:	*n* = 626	*n* = 625	*n* = 1251
Mean (SD)	7.1 (3.2)	7.3 (3.2)	7.2 (3.2)

^a^Index of multiple deprivation, 2015

^b^Based on UK 1990 reference centile curves and applying cut-offs used for population monitoring

^c^Not included in denominator for calculation of percentages

^d^Measured on a 5-point scale according to age-related expectations. Overall academic score is the sum of the ratings for math, reading and writing

The primary outcome is reported in Table 2. At 12 months (after start of intervention), an increase in mean BMIz from baseline was observed in both arms, and whilst the mean difference in BMIz indicates a smaller increase in the intervention compared with the control arm, this was not statistically or clinically significant, MD = −0.036, 95% CI −0.085 to 0.013, *p* = 0.146.

**Table 2:** Adjusted differences for body mass index (BMI) *z*-score between control and intervention groups at 4 and 12 months follow-up.

		Mean (SD) BMIz		Mean difference (95% CI), *P* value		Intra-cluster correlation coefficient^d^
Time point	No of participants^a^	Intervention arm	Control arm	Intervention v control (partial adjusted)^b^	Intervention v control (further adjusted)^c^	
Baseline		0.37 (1.25)	0.38 (1.25)			
4 months	Intervention *n* = 911 Control *n* = 732	0.42 (1.24)	0.42 (1.24)	−0.045 (−0.093 to 0.003), 0.07	−0.056 (−0.103 to −0.009), 0.02	0.005
12 months	Intervention *n* = 850 Control *n* = 820	0.45 (1.29)	0.47 (1.3)	−0.036 (−0.085 to 0.013), 0.15	−0.033 (−0.084 to 0.017), 0.20	0.001

^a^*n* for both partially and further adjusted analyses

^b^Adjusted for school size, % free school meals, school BMIz, school baseline outcome, participant baseline outcome

^c^Adjusted for school size, % free school meals, school BMIz, sex, ethnicity, deprivation (index of multiple deprivation score for school postcode), age, participant baseline outcome, school baseline outcome

^d^Estimates are unadjusted

Analysis of the secondary outcomes (Table 3) showed minor differences in favour of the intervention at 4 months for BMIz (MD −0.045, 95% CI −0.093 to 0.003) and body fat percentage (MD −0.35, 95% CI −0.79 to 0.08) which have minimal clinical relevance and were nevertheless greater than the differences at 12 months. The magnitude of effect changed slightly in the further adjusted models, but the direction of effect remained the same and most differences were statistically non-significant.

**Table 3:** Adjusted differences for all secondary outcomes between control and intervention arm at 4 and 12 months follow-up.

	Mean (SD)						Mean difference (95% CI), *P* value							
	Intervention arm			Control arm			Intervention vs control (partial adjusted)^a^				Intervention vs control (further adjusted)^b^			
Outcomes	Baseline	4 months	12 months	Baseline	4 months	12 months	4 months	*P* value	12 months	*P* value	4 months	*P* value	12 months	*P* value
Body fat %														
FU1 Intervention *n* = 911 Control *n* = 730 FU2: Intervention *n* = 844 Control *n* = 817	21.9 (7.1)	21.0 (7.3)	22.2 (7.8)	21.8 (6.9)	21.2 (7.2)	22.3 (7.7)	−0.35 (−0.79 to 0.08)	0.113	−0.43 (−0.87 to 0.00)	0.049	−0.18 (−0.61 to 0.24)	0.401	−0.01 (−0.42 to 0.40)	0.967
CHU-9D utility score^c^														
FU2 Intervention *n* = 980 Control *n* = 1015	0.83 (0.2)	Not collected	0.84 (0.2)	0.84 (0.2)	Not collected	0.83 (0.2)	N/A		0.003 (−0.05 to 0.05)	0.894	N/A		0.010 (−0.02 to 0.04)	0.500
MDI Wellbeing Index score														
FU2 Intervention *n* = 980 Control *n* = 1015	58.5 (10.9)	Not collected	59.1 (10.9)	57.8 (11.1)	Not collected	58.4 (11.5)	N/A		1.90 (−3.07 to 6.87)	0.499	N/A		0.56 (−2.15 to 3.27)	0.687

*CHU-9D* Child Health Utility 9 Dimensions instrument, *MDI* middle-years development instrument, *FU1* 4-month follow-up, *FU2* 12-month follow-up, *CI* confidence interval, *N/A* not applicable

^a^Adjusted for school size, % free school meals, school BMIz, school baseline outcome, participant baseline outcome

^b^Adjusted for school size, % free school meals, school BMIz, sex, ethnicity, deprivation (index of multiple deprivation score for school postcode), age, participant baseline outcome, school baseline outcome

^c^Measured on a zero to one scale with zero and one being a state equivalent to death and full health, respectively

There was a large amount of missing data for the other secondary outcomes. For fitness and academic attainment this exceeded 56% at certain time points and therefore multiple imputation was performed and both complete case and imputed variables are reported in Supplementary Appendix 2. For the complete case analysis, there was a small difference in academic attainment in favour of the intervention at 12 months (MD 1.36, 95% CI 0.62 to 2.10, *p* = < 0.001), but this was only statistically significant in the complete case analysis and not after imputation. For the measure of physical fitness (complete cases), there was a small difference in favour of the control group at both 4 (MD = −5.96, 95% CI 21.86 to 9.94, *p* = 0.436) and 12 months (MD = −65.51, 95% CI 113.81 to 17.21, *p* = 0.048) but this did not reach statistical significance for the imputed or complete case analysis. For wellbeing and quality of life, the maximum amount of missing data did not exceed 56% and although this is recognised as ‘high’, multiple imputation was performed. For these two secondary outcomes (quality of life and wellbeing) there were small, non-significant differences between groups in favour of the intervention (see Table 3). However, these results should be treated with caution and cannot be interpreted given the level of missingness.

In pre-specified subgroup analysis (see Table 4) there was significant interaction by sex with a modest and statistically significant intervention effect on BMIz for girls at 12 months in the partially-adjusted model (MD = −0.097, 95% CI −0.156 to −0.037, *p* = 0.001). There were also significant sex interactions with more favourable effects observed for bodyfat percentage (interaction test *p* = 0.001) in girls.

**Table 4:** Subgroup analysis^#^.

			Intervention		Control		Partially adjusted results*		Further adjusted results**	
			Baseline	Follow-up	Baseline	Follow-up				
			Mean (SD)	Mean (SD)	Mean (SD)	Mean (SD)	MD (95% CIs)	*p*	MD (95% CIs)	*p*
BMIz 12 months	Sex	Interaction test						0.007		0.004
		Male	0.452 (1.25)	0.533 (1.29)	0.391 (1.28)	0.494 (1.33)	0.021 (−0.046: 0.087)	0.542	0.027 (−0.040: 0.093)	0.434
		Female	0.285 (1.24)	0.356 (1.29)	0.369 (1.22)	0.456 (1.27)	−0.097 (−0.156: −0.037)	0.001	−0.094 (−0.158: −0.031)	0.003
	Year group (at baseline)	Interaction test						0.366		0.317
		Year 3	0.294 (1.23)	0.426 (1.25)	0.343 (1.23)	0.455 (1.27)	−0.015 (−0.082: 0.052)	0.654	−0.002 (−0.069: 0.065)	0.954
		Year 5	0.446 (1.26)	0.472 (1.33)	0.417 (1.27)	0.492 (1.33)	−0.056 (−0.122: 0.011)	0.100	−0.060 (−0.128: 0.009)	0.088
	IMD	Interaction test						0.155		0.123
		High deprivation	0.394 (1.25)	0.463 (1.30)	0.412 (1.26)	0.516 (1.29)	−0.054 (−0.109: 0.001)	0.054	−0.054 (−0.109: 0.000)	0.051
		Low deprivation	0.185 (1.19)	0.329 (1.20)	0.302 (1.21)	0.362 (1.31)	−0.149 (−0.437: 0.140)	0.312	−0.135 (−0.425: 0.154)	0.360
	Ethnic mix	Interaction test						0.235		0.187
		White	0.401 (1.22)	0.458 (1.26)	0.397 (1.20)	0.443 (1.27)	−0.015 (−0.082: 0.053)	0.673	−0.028 (−0.101: 0.045)	0.455
		Non-white	0.340 (1.28)	0.439 (1.32)	0.364 (1.30)	0.507 (1.33)	−0.070 (−0.135: −0.006)	0.032	−0.070 (−0.136: −0.005)	0.036
Bodyfat 12 months	Sex	Interaction test						0.001		<0.001
		Male	20.43 (7.12)	20.71 (7.78)	20.22 (6.59)	20.39 (7.58)	0.36 (−0.31: 1.03)	0.294	0.56 (−0.10: 1.22)	0.094
		Female	23.43 (6.63)	23.93 (7.39)	23.64 (6.71)	24.45 (7.34)	−0.70 (−1.17: −0.24)	0.003	−0.56 (−1.06: −0.07)	0.025
	Year group (at baseline)	Interaction test						0.190		0.453
		Year 3	21.58 (6.36)	22.12 (7.11)	21.66 (6.36)	22.06 (7.19)	0.05 (−0.44: 0.55)	0.828	0.30 (−0.15: 0.75)	0.190
		Year 5	22.10 (7.63)	22.33 (8.35)	22.01 (7.33)	22.59 (8.21)	−0.39 (−1.01: 0.23)	0.214	−0.26 (−0.86: 0.35)	0.407
	IMD	Interaction test						0.804		0.844
		High deprivation	21.96 (7.09)	22.35 (7.87)	22.00 (6.89)	22.43 (7.68)	−0.15 (−0.69: 0.39)	0.584	−0.17 (−0.71: 0.38)	0.553
		Low deprivation	20.90 (6.65)	21.12 (6.73)	21.43 (6.80)	22.04 (7.90)	−0.70 (−1.96: 0.56)	0.276	−0.44 (−1.70: 0.81)	0.491
	Ethnic mix	Interaction test						0.347		0.286
		White	21.48 (7.11)	21.84 (7.72)	21.30 (6.74)	21.66 (7.57)	−0.01 (−0.60: 0.57)	0.961	0.13 (−0.48: 0.74)	0.674
		Non-white	22.28 (6.97)	22.67 (7.81)	22.37 (6.95)	23.03 (7.85)	−0.41 (−0.98: 0.16)	0.158	−0.15 (−0.65: 0.34)	0.545

*Adjusted for school size, % free school meals, school BMIz, school baseline outcome, participant baseline outcome

**Adjusted for school size, % free school meals, school BMIz, sex, ethnicity, deprivation (index of multiple deprivation score for school postcode), age, participant baseline outcome, school baseline outcome

^#^Subgroup analysis not conducted on fitness, academic attainment, wellbeing or quality of life due to missing data (≥56%)

In the further adjusted model, the mean intervention effect on QALYs was 0.006 (95% CI: −0.005 to 0.018, *p* = 0.25). The additional costs for The Daily Mile schools were due to the staff cost from supervising the intervention. There were no costs attributable to the control arm so the further-adjusted mean difference in cost was £48.33 (95% CI: £48.21–£48.25) per child. Offsetting the additional per child cost of the intervention alongside the QALY-gain, results in an ICER of £7,455.21 per QALY gained, which is well within the range of what is conventionally regarded as cost-effective within a UK setting. A net benefit framework was applied to assess the level of decision uncertainty represented using a cost-effectiveness acceptability curve that plots the probability that the intervention is cost-effective at different levels of willingness to pay. At the UK threshold of £20,000 per QALY, the probability that the intervention is cost-effective was 76%.

The economic analyses was conducted separately for boys and girls and is reported in full within the Supplementary Appendix 1. In summary, it indicates The Daily Mile to be highly cost-effective in girls with a statistically significant gain in QALYs (independent of costs), an ICER of £2,492 per QALY, and a 97% probability of cost-effectiveness at the UK threshold of £20,000 per QALY. For boys, the economic result was different as The Daily Mile had only a 12% chance of cost-effectiveness when compared with usual activities. This is because boys incurred a (non-significant) loss in QALYs (−0.007, 95% CI: −0.021 to 0.008).

## Discussion

We found that The Daily Mile intervention did not have a clinically important effect on BMIz overall. There was however evidence of a modest intervention effect on BMIz for girls with consistent findings for body fat percentage. The economic analysis also indicates the intervention to be highly cost-effective in girls, and in the whole sample, there is a 76% chance of cost-effectiveness using standard UK thresholds. Overall, or within subgroups, there was no evidence of an effect on wellbeing.

Our findings differ from those reported by a small pilot study of The Daily Mile in two Scottish schools [12], which showed significant improvement in fitness and lower adiposity assessed by sum of skinfolds in the intervention school, but no difference in BMIz. However, lack of randomised allocation, inclusion of only two clusters and absence of a prior published protocol limit interpretation. Reviews of previous school-based physical activity interventions have had mixed results. Although a Cochrane review of 44 trials did not find any overall effect on adiposity [8], a more recent review of 18 studies assessing longer term outcomes (>12 months) showed a small overall effect of school-based physical activity interventions on reducing gains in adiposity [33]. Both reviews identified a need for further high-quality studies. More broadly, our findings also differ from those reported by more recent school-based childhood obesity prevention studies in the UK that included a physical activity element [34, 35]. These trials did not find evidence of multicomponent interventions having any impact on reducing BMIz or obesity.

This is the first cluster RCT of The Daily Mile. The design was pragmatic, conducted over a 12-month period to accommodate usual school vacation time and to generate rapid results to inform global policy action. The trial protocol was published in advance. Baseline measurements were collected prior to randomisation and all statistical analysis took account of clustering. Furthermore, the study contained an economic evaluation, using instruments validated specifically for use within school-aged children.

There were some limitations. Whilst missing data are low for the primary outcome (BMIz) and body fat percentage, it was high (56%) for two secondary outcome measures (quality of life and wellbeing) and very high (>56%) for other secondary outcome measures (fitness and academic attainment), which meant these are more difficult to interpret and reported in the appendix. This was attributable to the time commitment required to collect these data by schools. Research staff obtained anthropometric measures, whereas fitness, academic attainment and wellbeing measures were administered by school staff.

The pragmatic nature of the study was both a strength and a weakness. The schools were provided with minimal training and advised to implement The Daily Mile. There was little interference from the research staff, and schools were autonomous in their implementation. Our findings therefore reflect how it would be administered outside of a research setting. Whilst a formal process evaluation was not conducted, interviews with school staff indicated that The Daily Mile was largely not conducted daily, and implementation fluctuated depending on competing demands during the school year. The timing of data collection might also have had a bearing on the study findings. Mean difference in BMIz at 4 months was statistically significant and larger than at 12 months, though the magnitude of difference may not be clinically important. This might suggest that The Daily Mile is initially beneficial, but the intervention effect does not endure or could be due to seasonal variations in weight between winter and summer. Maintaining staff and children’s motivation could be important for The Daily Mile to provide long-term benefits. Whilst providing rapid evidence of an important and policy-relevant question, longer-term follow up would provide insight into the longitudinal effect of The Daily Mile on children’s health and wellbeing and its long-term cost-effectiveness.

The minimal effect on BMIz observed in the whole sample may be a result of a lack of compliance. It is perhaps unrealistic for schools to complete The Daily Mile every day, given the competing activities and focus on academic attainment. Furthermore, The Daily Mile only addresses one aspect of the energy imbalance that contributes to excess weight. Multi-component childhood obesity interventions that address both physical activity and dietary behaviours are likely to be more effective [36], with those also involving home elements being the most successful [37]. There may be several explanations for the observed differential intervention benefits in girls. Boys are generally less sedentary than girls during school hours, providing less potential for change. This sex difference is not unusual, and similar findings have been reported in other school-based RCTs of combined physical activity and education interventions [38, 39]. This study was not powered to detect this subgroup effect, which could be a consideration in further studies. The exploratory economic results are interesting as although the intervention was found to be highly cost-effective in girls, it was ‘dominated’ in boys which means that when compared with usual activities, The Daily Mile both cost more and led to a QALY-loss (in boys). This QALY-loss for boys was not statistically significant at the 5%-level raising an interesting normative question of whether society should be willing to pay for a universal intervention that is potentially highly cost-effective in girls but not in boys, but causes no ‘harm’ in boys. Furthermore, it should be noted that the economic analysis was exploratory as there were high levels of missing CHU9D data; and it was conservative as it did not account for any potential future cost-savings from having a modest intervention effect for girls.

At a time when childhood obesity is a major problem worldwide, schools have been identified as a key place to improve physical activity levels. This study has shown that interventions like The Daily Mile are not likely to have an effect on BMIz overall but within girls, it could be highly cost-effective. The study finding that BMIz increased in both groups over 12 months highlights the critical nature of childhood obesity and puts the emphasis on urgent action. Whilst interventions such as The Daily Mile are not going to reduce childhood obesity alone, they should be considered as part of a whole system approach to childhood obesity prevention [40–42].

## Supplementary information

Supplementary Appendix
