# Supplementary material for: Effectiveness and cost-effectiveness of The Daily Mile on childhood weight outcomes and wellbeing: a cluster randomised controlled trial
Source: Int J Obes (Lond). 2020 Jan 28;44(4):812–22. doi: 10.1038/s41366-019-0511-0 (PMC7101281; doi:10.1038/s41366-019-0511-0)
Supplement: Supplementary file 1 — Supplementary Appendix [file 41366_2019_511_MOESM1_ESM.docx]

**Supplementary Appendix 1: Exploratory Economic Evaluation**

**Complete case analysis**

There was a high proportion of missing data for the economic outcome (CHU9D, missing data = 47%) and therefore the economic analysis was from a complete case analysis as it was felt that the proportion of missing CHU9D data was too high to justify multiple imputation. The economic evaluation therefore is exploratory in nature and the results should be interpreted with caution.

The characteristics of the sample included in the economic evaluation are reported in Table S1. The sample was similar to the clinical outcome dataset and characteristics appeared balanced between arms.

Table S1 Economic evaluation sample characteristics

|  |  | Intervention | Control |
| --- | --- | --- | --- |
| N |  | 461 | 327 |
| Child Age in Years, mean (SD) | | 8.89 (1.04) | 8.9 (1.00) |
| Sex N (%) | Female | 220 (47.7) | 156 (47.7) |
|  | Male | 241 (52.3) | 171 (52.3) |
| Ethnicity | White | 288 (62.5) | 166 (51.2) |
|  | South Asian | 68 (14.8) | 49 (15.1) |
|  | Black African Caribbean | 31 (6.7) | 37 (11.4) |
|  | Other/not specified | 74 (16.1) | 72 (22.2) |
| IMD Quintile | 1 | 260 (56.4) | 156 (47.7) |
|  | 2 | 152 (33.0) | 103 (31.5) |
|  | 3 | 0 (0.0) | 59 (18.0) |
|  | 4 | 49 (10.6) | 9 (2.8) |
| Year group | Year 3 | 223 (48.4) | 151 (46.2) |
|  | Year 5 | 238 (51.6) | 176 (53.8) |
| Baseline BMI z-score, mean (SD) | | 0.409 (1.25) | 0.383 (1.27) |
| Baseline body fat, mean (SD) | | 22.03 (7.16) | 21.93 (6.96) |

**Methods for measuring resource use and costs**

Costs included in the economic evaluation were the teacher time to conduct the Daily Mile. Costs reported are the costs to conduct the Daily Mile for an average child for 12 months. Average local authority primary school teacher’s salary (1) was calculated over a 1265 hour school year (2) and are reported in table S1. To calculate the intervention costs, it was assumed that the Daily Mile would be implemented 5-days a week for 15 minutes per day, based on data derived from interviews with school staff.

Table S2: Unit costs and Resource Use

|  | Detail/Assumption | Unit Cost (£) | Source |
| --- | --- | --- | --- |
| School teacher’s time | Average annual salary  Average hourly salary assuming 1265 hour working school year (2) | 34,300  27.11 | (1) |
| Intervention Costs |  |  |  |
| Teacher time | Cost of 15-minutes of teacher’s time  Average intervention cost per child (assuming average class size of 27 children and a 190 day school year (3)) | 6.77  47.53 |  |

The average intervention cost per child was derived assuming an average primary school class size of 27 children (4). The cost of the comparator arm for usual activities was assumed to be zero.

**Methods for the economic evaluation**

The aim of the economic evaluation was to assess the cost-effectiveness of the Daily Mile, compared with usual school activities for effecting child weight outcomes in primary schools, and to conduct a cost-utility analysis using the incremental cost per Quality Adjusted Life Year (QALY) gained over the 12-month time period of the trial. Both the cost-effectiveness and the cost-utility analysis were within trial analysis and therefore only considered the data collected over the 12-month duration of the trial, discounting on either costs or outcomes was therefore not conducted. All analyses were from a public sector perspective, including costs to the schools only and are reported in 2017 prices.

The generic preference-based CHU9D (5) was used to estimate utility values using the UK tariff (6) and QALYs were generated using the area under the curve method (7). As noted previously, the amount of missing data was deemed high but given the importance of generating economic evidence and with the emphasis on QALY’s as an outcome that is used within a UK and international decision-making context, a decision was made to proceed with an exploratory analysis.

To conduct the economic evaluation, the difference in costs between arms were estimated using general linear mixed models, adjusted for variables used in randomisation (percentage of children receiving free school meals, school size and school baseline BMIz), clustering (school). Differences in QALYs were analysed used hierarchical linear regression, also adjusting for the aforementioned factors, participant characteristics (age, sex, ethnicity and IMD) and baseline utility. The mean difference in cost was divided by the mean difference in QALYs, to produce an incremental cost-effectiveness ratio (ICER) and therefore the cost per additional QALY gained for having the Daily Mile versus a situation when no Daily Mile is in place. As well as the analyses being conducted on the whole sample, a separate analysis was conducted by sex due to the underlying evidence that girls become more sedentary in behaviour as they approach adolescence (8, 9).

To estimate the uncertainty around the ICER, a probabilistic sensitivity analysis was applied using a method called bootstrapping. Bootstrapping empirically constructs 1000 paired cost/QALY estimates and allows a graphical representation of the pairs of cost/QALY ratios using a 4-quadrant diagram. A cost-effectiveness acceptability curve (CEAC) can then be constructed using the plotted points and a net-monetary approach (7). The CEAC shows the probability of the Daily Mile being cost-effective at difference cost per QALY thresholds. In the UK, interventions are deemed cost effective if the cost per additional QALY gained is less than £20,000 per QALY (10).

All analyses were conducted in STATA (Version 13).

**Results of the economic evaluation**

Table S3 reports utility scores calculated at baseline and 12-month follow-up. The mean costs and QALYs and testing of differences are reported in Table S4. Children in the intervention arm accrued more QALYs than those in the usual activities arm (mean difference 0.006), although this difference was not significant (p=0.252). The mean cost of the Daily Mile was £45.44, with the intervention costing an additional £48.33 per year per child per school year, compared to usual activities after adjustment for baseline differences and covariates. This was significant (p=0.00). The ICER was £7,445.21 per incremental QALY gained.

Table S3 Utilities calculated using the CHU9D

|  | Intervention | Control |
| --- | --- | --- |
| Baseline utility  Mean (SD) | 0.833 (0.165) | 0.842 (0.157) |
| Follow-up utility  Mean (SD) | 0.840 (0.152) | 0.835 (0.156) |

Table S4 Analyses of costs and outcomes on whole sample

|  | Intervention | | Control | |  |  |  |  |
| --- | --- | --- | --- | --- | --- | --- | --- | --- |
|  | Mean | SD | Mean | SD | MD* (95% Lower CI: Upper CI) | P-value | ICER (£) |  |
| QALYs | 0.836 | 0.134 | 0.839 | 0.13 | 0.006 (-0.005: 0.018) | 0.252 | 7,455.21 |  |
| Cost per child (£) | 45.44 | 5.67 | 0 | 0 | 48.33 (48.21: 48.45) | 0.00 |  |  |
| MD – Mean difference SD – Standard Deviation ICER- Incremental cost-effectiveness ratio CI= confidence interval | | | | | | | | |

Table S5 Analyses of costs and outcomes on girls

|  | Intervention | | Control | |  |  |  |
| --- | --- | --- | --- | --- | --- | --- | --- |
|  | Mean | SD | Mean | SD | MD* (95% Upper CI: Lower CI) | P-value | ICER (£)  (95% Lower CI: Upper CI) |
| QALYs | 0.840 | 0.13 | 0.826 | 0.13 | 0.192 (0.002: 0.036) | 0.024 | 2,492.38  (1,244.31:3,785.94) |
| Cost per child (£) | 45.34 | 5.79 | 0 | 0 | 47.85 (47.71: 48.01) | 0.00 |  |
| MD – Mean difference SD – Standard Deviation ICER- Incremental cost-effectiveness ratio CI= confidence interval | | | | | | | |

Table S6 Analyses of costs and outcomes on boys

|  | Intervention | | Control | |  |  |  |
| --- | --- | --- | --- | --- | --- | --- | --- |
|  | Mean | SD | Mean | SD | MD* (95% Lower CI: Upper CI) | P-value | ICER (£) |
| QALYs | 0.833 | 0.14 | 0.850 | 0.12 | -0.007 (-0.021: 0.008) | 0.361 | -6,932.97 |
| Cost per child (£) | 45.453 | 5.56 | 0 | 0 | 47.08 (46.92: 47.23) | 0.00 |  |
| MD – Mean difference SD – Standard Deviation ICER- Incremental cost-effectiveness ratio CI= confidence interval | | | | | | | |

For the whole sample, when the cost and QALY differences were combined, they showed the Daily Mile to be cost-effective which means the intervention cost an additional £7,455 per QALY which is well below the standard UK threshold value of £20,000 per QALY. To assess the uncertainty, the cost-effectiveness plane (Figure S1) presents the 1,000 jointly bootstrapped cost-QALY pairs distributed across four quadrants. Most of the pairs lie in the north-east quadrant indicating that the Daily Mile produces QALY gains at an additional cost, however 14% of the cost-effectiveness plots fell in the north-east quadrant indicating a loss in QALYs.

When the analyses were conducted separately for boys and girls some interesting results emerge. There was a clear difference in cost-effectiveness for boys and girls. For boys, the majority (84%) of bootstrapped cost-QALY pairs are in the north-west quadrant, this means that the Daily Mile is both cost-incurring and leads to a loss in QALYs, when compared to usual activities. In economics terms this result is referred to as being ‘dominated’, as the intervention (the Daily Mile) both costs more and leads to a loss in outcome, when compared to usual activities. If the effect on outcome is analysed independently (without considering cost) for boys, although there is a loss in QALYs, this loss is not statistically significant at the 5% level – see Table S6. For girls, the opposite is the case with the majority of cost-effect pairs being in the north-east quadrant which means the Daily Mile leads to a gain in QALYs and is cost-incurring. When outcome is considered independently for girls – see Table S5 – this positive effect is statistically significant at the 5% level.

The CEAC reflects the decision uncertainty surrounding the choice of implementing the Daily Mile over usual activities (Figure S4) and shows a 76% chance of cost-effectiveness for the whole sample, and a 97% and 12% chance of cost-effectiveness for girls and boys, respectively, at the commonly applied UK threshold value of £20,000 per QALY.

**Figure S1: Cost-utility plane for Daily Mile versus Usual Activities – whole sample (based on 1,000 bootstrapped cost-effect pairs)**


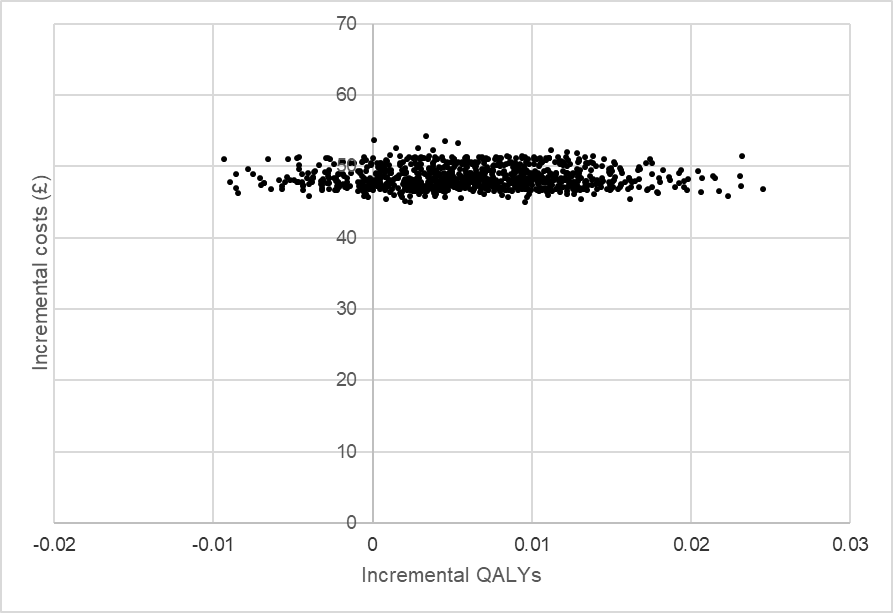


**Figure S2: Cost-utility plane for Daily Mile versus Usual Activities – girls (based on 1,000 bootstrapped cost-effect pairs)**


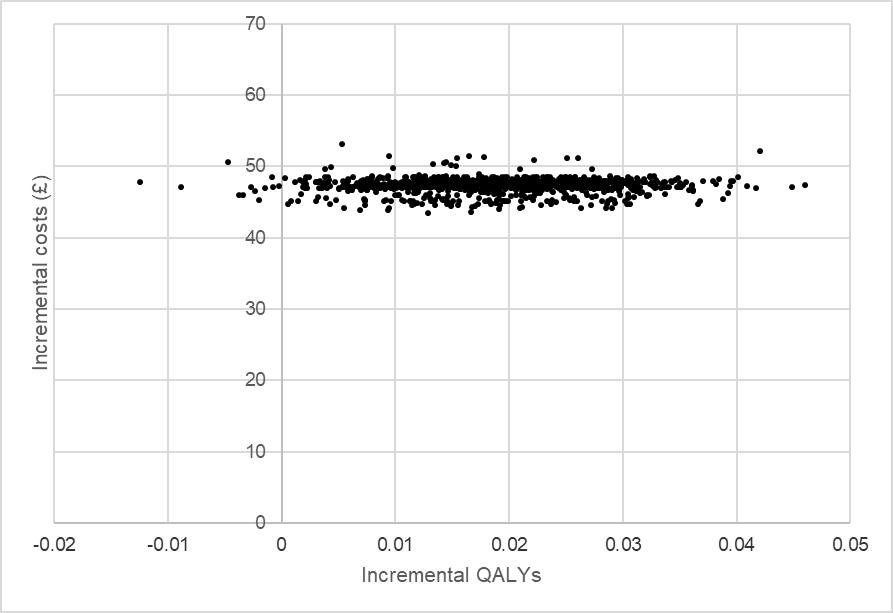


**Figure S3: Cost-utility plane for Daily Mile versus Usual Activities – boys (based on 1,000 bootstrapped cost-effect pairs)**


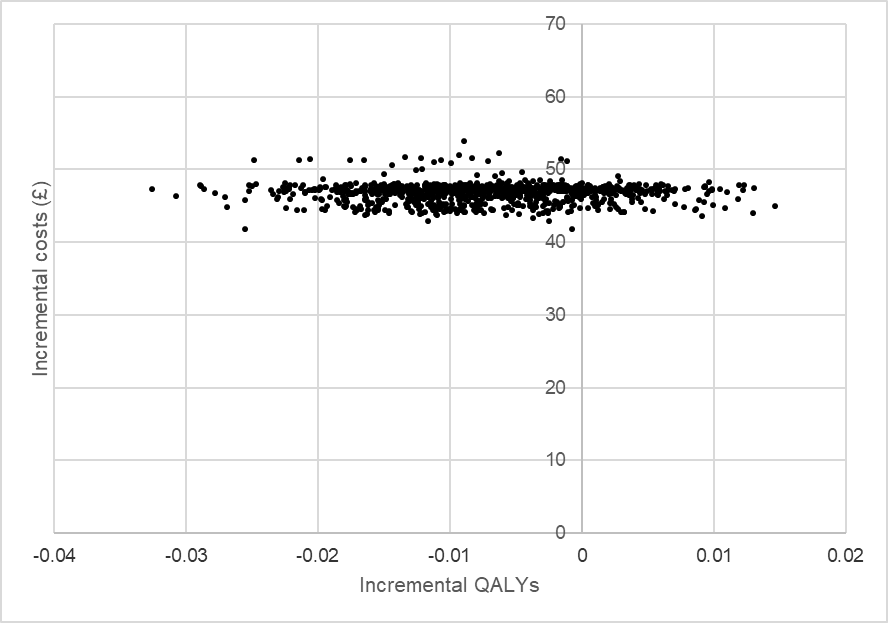


**Figure S4: Cost-utility acceptability curve comparing Daily Mile and usual activities for whole sample, girls and boys (based on 1,000 bootstrapped cost-QALY pairs)**


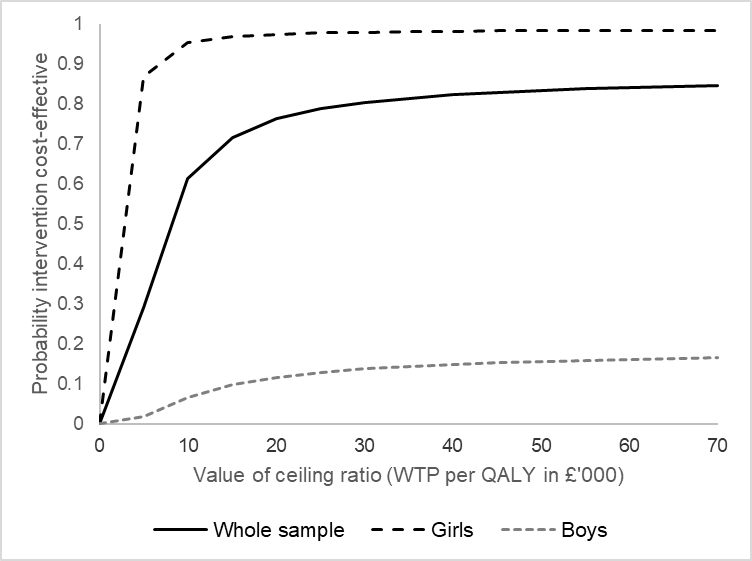


**References for Supplementary Appendix 1**

1. Department for Education. School workforce in England. . 2017.

2. Department for Education. School teachers’ pay and conditions document 2017 and guidance on school teachers’ pay and conditions. 2017.

3. Department for Education. Education (School Day and School Year) (England) Regulations 1999.

4. Department for Education. Schools, pupils and their characteristics: January 2017. 2017.

5. Stevens K. Developing a descriptive system for a new preference-based measure of health-related quality of life for children. Quality of life research : an international journal of quality of life aspects of treatment, care and rehabilitation. 2009;18(8):1105-13.

6. Stevens K. Valuation of the Child Health Utility 9D Index. Pharmacoeconomics. 2012;30(8):729-47.

7. Glick HA, Doshi JA, Sonnad SS, Polsky D. Economic evaluation in clinical trials: OUP Oxford; 2014.

8. Kimm SYS, Glynn NW, Kriska AM, Barton BA, Kronsberg SS, Daniels SR, et al. Decline in Physical Activity in Black Girls and White Girls during Adolescence. New England Journal of Medicine. 2002;347(10):709-15.

9. Wolf AM, Gortmaker SL, Cheung L, Gray HM, Herzog DB, Colditz GA. Activity, inactivity, and obesity: racial, ethnic, and age differences among schoolgirls. Am J Public Health. 1993;83(11):1625-7.

10. NICE. Developing NICE Guidelines: The Manual. 2014.

**Supplementary Appendix 2: Adjusted differences for Academic Attainment and Fitness between control and intervention arm at 4- and 12- month follow-up: Complete case and Imputed Values**

Table S7: Imputed Values

|  | Mean (SD) | | | | | | Mean difference (95% CI), P value | | | | | | | |
| --- | --- | --- | --- | --- | --- | --- | --- | --- | --- | --- | --- | --- | --- | --- |
|  | Intervention arm | |  | Control arm | | | Intervention v control (partial adjusted)* | | | | Intervention v control (further adjusted)** | | | |
| Outcomes | Baseline | 4 months | 12 months | Baseline | 4 months | 12 months | 4 months | P value | 12 months | P value | 4 months | P value | 12 months | P value |
| Linear track test (metres)^φ^ |  |  |  |  |  |  |  |  |  |  |  |  |  |  |
| FU1  Intervention n=850  Control n=646  FU2  Intervention n=825  Control n=686 | 350.7 (105.1) | 353.6 (71.9) | 296.3 (69.6) | 345.9 (81.2) | 370.9 (90.3) | 328.6  (99.6) | -15.3  (-38.2 to 7.63) | 0.191 | -37.4  (-73.0 to -1.67) | 0.040 | -20.0 (-40.7 to 0.71) | 0.058 | -37.4 (-74.7 to -0.19) | 0.049 |
| Academic attainment |  |  |  |  |  |  |  |  |  |  |  |  |  |  |
| FU2  Intervention n=754  Control=735 | 7.1 (3.2) | Not collected | 8.4 (3.3) | 7.3 (3.3) | Not collected | 7.6 (3.0) | N/A |  | -0.04 (-1.44 to 1.37) | 0.955 | N/A |  | 0.19 (-1.12 to 1.51) | 0.773 |

Table S8: Complete Case Values

|  | Mean (SD) | | | | | | Mean difference (95% CI), P value | | | | | | | |
| --- | --- | --- | --- | --- | --- | --- | --- | --- | --- | --- | --- | --- | --- | --- |
|  | Intervention arm | |  | Control arm | | | Intervention v control (partial adjusted)* | | | | Intervention v control (further adjusted)** | | | |
| Outcomes | Baseline | 4 months | 12 months | Baseline | 4 months | 12 months | 4 months | P value | 12 months | P value | 4 months | P value | 12 months | P value |
| Linear track test (metres)^φ^ |  |  |  |  |  |  |  |  |  |  |  |  |  |  |
| FU1  Intervention n=129  Control n=312  FU2  Intervention n=296  Control n=552 | 350.7 (105.1) | 353.6 (71.9) | 296.3 (69.6) | 345.9 (81.2) | 370.9 (90.3) | 328.6  (99.6) | -3.01  (-21.25 to 15.22) | 0.746 | -59.79  (-105.80 to -13.78) | 0.011 | -5.96 (-21.86 to 9.94) | 0.463 | -65.51 (-113.81 to -17.21) | 0.008 |
| Academic attainment |  |  |  |  |  |  |  |  |  |  |  |  |  |  |
| FU2  Intervention n=351  Control=588 | 7.1 (3.2) | Not collected | 8.4 (3.3) | 7.3 (3.3) | Not collected | 7.6 (3.0) | N/A |  | 1.16 (0.53 to 1.78) | 0.000 | N/A |  | 1.36 (0.62 to 2.10) | 0.000 |

FU1=4-month follow-up FU2=12-month follow-up CI=confidence interval N/A=not applicable

^φ^ Number of metres ran in two-minute track test.

^##^ Sum score based on age-related expectation scores from one to five for math, reading and writing.

*Adjusted for school size, % free school meals, school BMIz, school baseline outcome, participant baseline outcome.

**Adjusted for school size, % free school meals, school BMIz, sex, ethnicity, deprivation (index of multiple deprivation score for school postcode), age, participant baseline outcome, school baseline outcome.
